# *BCR-ABL* Gene Transcript Types of Patients with Chronic Myelogenous Leukemia in Yogyakarta, Indonesia

**DOI:** 10.31557/APJCP.2020.21.6.1545

**Published:** 2020-06

**Authors:** Dewi Kartikawati Paramita, Susanna Hilda Hutajulu, Anditta Syifarahmah, Tri Agusti Sholika, Sri Fatmawati, Sumartiningsih Aning, Dewi Sulistyawati, Sri Wahyuni, Kartika Widayati Taroeno-Hariadi, Johan Kurnianda

**Affiliations:** 1 Department of Histology and Cell Biology, Faculty of Medicine, Public Health and Nursing, Universitas Gadjah Mada, Yogyakarta, Indonesia.; 2 Molecular Biology Laboratory (Integrated Research Laboratory), Faculty of Medicine, Public Health and Nursing, Universitas Gadjah Mada, Yogyakarta, Indonesia.; 3 Division of Hematology and Medical Oncology, Department of Internal Medicine, Faculty of Medicine, Public Health and Nursing, Universitas Gadjah Mada/ Dr Sardjito General Hospital, Yogyakarta, Indonesia.; 4 Medical Study Program, Faculty of Medicine, Public Health and Nursing, Universitas Gadjah Mada, Yogyakarta, Indonesia.; 5 Basic Medical Science Study Program, Faculty of Medicine, Public Health and Nursing, Universitas Gadjah Mada, Yogyakarta, Indonesia.

**Keywords:** Chronic myelogenous leukemia (CML), BCR-ABL, b3a2, b2a2, major breakpoint

## Abstract

**Background:**

The aim of this study was analyzing the BCR-ABL transcript types of patients with chronic myeloid leukemia (CML) in Dr Sardjito General Hospital, Yogyakarta, Indonesia. This study is very relevant because the data concerning *BCR-ABL* gene transcript types is very limited in Indonesia. Furthermore, it is important for patient’s management, particularly in defining the tyrosine kinase inhibitors (TKIs) therapy and monitoring after therapy. The introduction of TKIs has become a major advance in the management of patients with CML, especially in the chronic phase (CML-CP), in which most patients are diagnosed.

**Methods:**

One hundred eighty five (185) of 370 recruited patients were included in this study (2010–2014). RNA samples were isolated from mononuclear cells of peripheral blood of the subjects taken at primary diagnosis. Detection of *BCR-ABL* gene transcript types was done using multiplex reverse transcriptase PCR (multiplex RT-PCR) and/or nested PCR following the cDNA synthesis. When the first PCR set failed to amplify the *BCR-ABL* gene, RT-conventional PCR and/or nested PCR would be applied. The proportion of each transcript type was calculated among the BCR-ABL positive CML patients.

**Results:**

Approximately 99% (183/185) of CML patients are BCR-ABL positive, with the most common type is major b3a2 (136/183; 74.3%), followed by major b2a2 (41/183; 22.4%). Two samples (1.1%) showed co-expression of b3a2 and b2a2; 1 sample showed co-expression of b3a2 and fragment at 500bp; and 3 samples showed uncommon fragments.

**Conclusion:**

Ninety nine percent (99%) of CML patients in Yogyakarta, Indonesia are BCR-ABL positive, with 74.3% have b3a2 transcript, 22.4% have b2a2 trascript, 1.1% have co-expression of b3a2 and b2a2 transcript, and the rest (2.2%) have uncommon bands that still need to be confirmed.

## Introduction

Chronic myelogenous leukemia (CML) is a clonal malignancy of pluripotent hematopoietic stem cells, characterized by a cytogenetic abnormality known as Philadelphia (Ph) chromosome. The Ph chromosome occurs due to a reciprocal translocation between chromosomes 9 and 22 that generate the *BCR-ABL* fusion gene. The BCR-ABL encodes protein with aberrant ABL tyrosine kinase activity, which plays an essential role in CML pathogenesis (Deininger et al., 2000; Kantarjian et al., 2006; Jabbour et al., 2008; Chen et al., 2010; Alikian et al., 2017; Gale and Apperley, 2019). The important identification of the elevation of tyrosine kinase activity in *BCR-ABL* fusion gene in CML pathogenesis contributed in developing effective tyrosine kinase inhibitors (Druker, 2008).

Chronic myelogenous leukemia is the most common leukemia in Asia (Anand et al., 2012), including in Indonesia, corresponds to 15-20% of total leukemia patients (Handayani and Sulistyo, 2008). Data on patients with CML in Indonesia is very limited, especially the molecular and genetic aspects, including the primary data on the breakpoint type of *BCR-ABL* gene transcripts. Whereas, understanding the molecular basis of CML provides important information in the development of diagnosis and monitoring methods in response to therapy and disease recurrence (Baccarani et al., 2006; Jabbour et al., 2008). The introduction of tyrosine kinase inhibitors improves the management of patients with CML, especially in the chronic phase (CML-CP) (Marin et al., 2012). 

The BCR-ABL breakpoint type in CML is highly correlated to the clinical course and outcome of the patients. In general, three breakpoint cluster regions in BCR gene have been characterized, M-bcr (major-bcr), m-bcr (minor-bcr) and μ-bcr (micro-bcr) (Yaghmaie et al., 2008; Alikian et al., 2017). Due to alternative splicing, two major breakpoints are known as b3a2 and b2a2, and both mRNAs are translated into p210 BCR-ABL protein (Yaghmaie et al., 2008; Mir et al., 2015), and contribute to most of the classical CML phenotype. Minor breakpoint cluster region (m-bcr) are translated into p190 BCR-ABL protein and μ-bcr giving rise to p230 BCR-ABL protein and frequently manifest in chronic neutrophilic leukemia (Yaghmaie et al., 2008). Identification and quantification of Ph chromosomes in bone marrow are very important in confirming diagnosis and monitoring the response to therapy. The response of specific therapy such as allogeneic stem cell transplantation, interferon-α, and tyrosine kinase (TK) inhibitors can be assessed by quantification of BCR-ABL transcript. The monitoring of Ph chromosome in the patients undergoing these therapeutic approaches can predict whether the patients have higher risk of disease progression (Hughes et al., 2006), which is indicated by the accumulation of molecular abnormalities that cause loss of the capacity for terminal differentiation of the leukemic clone (Calabretta and Perrotti, 2004). 

Previous publications reported the frequency of BCR-ABL breakpoint type in many countries (Paz-y-Miño et al., 2002; Anand et al., 2012; Hanfstein et al., 2014), but none of the data from Indonesia was reported. The incidence of CML is different from one country to another, and the frequency of BCR-ABL breakpoint types is different among ethnic backgrounds (Anand et al., 2012; Hanfstein et al., 2014; Paz-y-Miño et al., 2002). Several molecular methods have been developed to detect the BCR-ABL transcript, but RT-PCR is considered as the most sensitive one. In this study using RT-PCR, we analyzed the BCR-ABL breakpoint types of the CML patients in Dr Sardjito General Hospital, Yogyakarta, Indonesia.

## Materials and Methods

*Study subjects*

Three hundred seventy (370) CML patients were recruited prospectively during March 2010 to December 2014 at Dr Sardjito General Hospital, Yogyakarta, Indonesia, at first visit prior to any therapeutic treatment. One hundred eighty five (185) of 370 patients with complete medical record data were included in this study. 

Ten (10) ml peripheral blood samples were taken from those 185 patients using EDTA vacutainer. This study was approved by The Ethical Committee of Faculty of Medicine, Public Health and Nursing, Universitas Gadjah Mada/Dr Sardjito General Hospital, Yoyakarta, Indonesia (No. KE/KF/1029/EC). 

*RNA extraction and cDNA synthesis*

RNA was extracted from mononuclear cells separated from peripheral blood. RNA extraction was performed by standard protocol using Trizol. The RNA quantity and quality was assessed using GeneQuant II (Pharmacia Biotech), and the OD ratio of 260/280 should be at least 1.8. One gram of total RNA was used for reverse transcription using Transcriptor First Strand cDNA Synthesis Kit (Roche; Cat no. 04 897 030 001). The cDNA synthesis was carried out at 25°C for 10 min and continued at 42°C for 60 min. The reaction to inactivate the transcriptor reverse transcriptase was done at 99^o^C for 5 minutes and the cDNA stored at -20^o^C until used. 

*BCR-ABL transcript Detection*

The initial detection of BCR-ABL transcript in patients with CML was performed by using multiplex RT-PCR and/or in combination with nested RT-PCR. When the amplification using those methods failed to amplify the transcript, it was continued using conventional and/or a combination of methods with nested RT-PCR. 

*Multiplex and nested PCR*

The detection of *BCR-ABL* gene transcript fragments were initially detected using multiplex PCR, which allowed amplifying all of the breakpoint types. When the multiplex PCR failed to amplify the transcript, it was continued to the nested PCR using the product of multiplex PCR as the template. The multiplex and nested PCR was performed according to previous study (Goh et al., 2006; Sholikah et al., 2017). The product for multiplex PCR should be 627bp for b3a2; 552bp for b2a2; 378bp for b2a3; 580bp for b1a1; 429bp for e1a2; 1167bp for c3a2; and 255bp for e1a3. The product for nested PCR should be 443bp for b3a2; 368bp for b2a2; 194bp for b2a3; 396bp for b1a1; 378bp for e1a2; 983bp for c3a2; and 204bp for e1a3.

*Conventional PCR*

Several samples that gave negative result in multiplex and nested PCR were re-amplified using conventional PCR and/or nested PCR. The conventional PCR was initially done using primers for major breakpoint and when it gave negative result, the product was used as the template for nested PCR using internal primers. When the PCR for major breakpoint did not give positive result, the conventional and/or nested PCR using primers for both minor and micro were performed. The conventional and nested PCR were performed according to previous publication (Goh et al., 2006; Sholikah et al., 2017), with modification of annealing temperature at 63OC for 1st round PCR. The product for conventional PCR should be 616bp for b3a2 and 552bp for b2a2 and for nested PCR should be at 443bp for b3a2 and 368bp for b2a2. 

**Figure 1:**
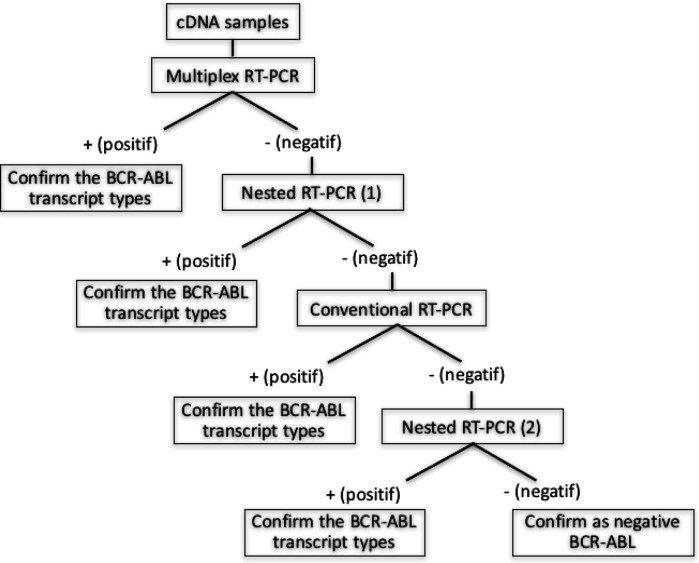
Algorithm of BCR-ABL Transcript Type Detection. Initially, cDNA samples were amplified by using multiplex RT-PCR. This multiplex PCR can amplify all of the breakpoint types of BRC-ABL transcript. The ones gave negative result would be amplified using nested RT-PCR for major breakpoint. The samples that still failed to be amplified previously were amplified using conventional RT-PCR using primers for major breakpoints. The ones gave positive result confirmed as major breakpoint type either b3a2 or b2a2 or combination of b3a2 and b2a2. The samples with negative result in the conventional PCR will be used as template for nested RT-PCR using primers for major breakpoints. The positive samples confirmed as major breakpoint and the negative ones confirmed as CML with negative BCR-ABL

The algorithm of BCR-ABL transcript type detection in this study can be seen in Figure1. 

## Results

**Figure 2:**
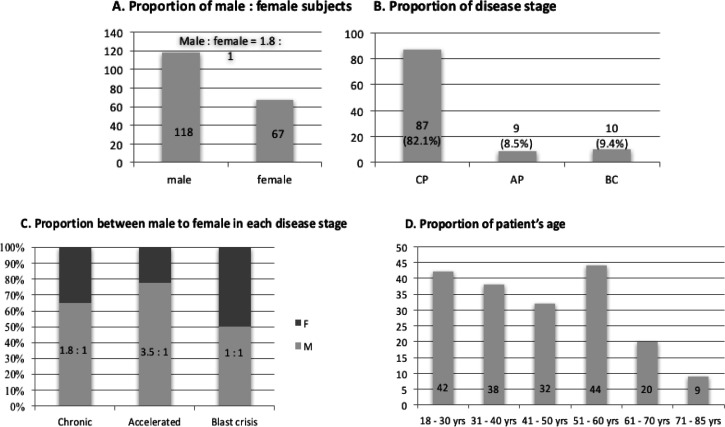
Characteristic of the Subjects. (A), Proportion of male and female subjects. The number of male subjects in this study was 118 and the female was 67, with ratio of male and female was 1.8:1; (B), Proportion of disease stage. Most of the patients were in chronic phase (82.1%), continued with blast crisis (9.4%) and accelerated phase (8.5%); (C), The Proportions between male to female in each disease stages. The ratios between male to female are 1.8, 3.5 and 1 in CP, AP and BC respectively; (D), Proportions of patient’s age

One hundred eighty five (185) patients with relatively complete data in medical records were included in this study, and showed 1.8:1 male and female ratio (Figure 2A). One hundred eighty three (183) of 185 patients are positive for BCR-ABL, therefore only 183 patient’s data were further analyzed. The stage of CML in patient’s data was only available for 106 of 183 patients. Most of the patients are in the chronic phase (CP) (87/106; 82.1%); followed by blast crisis, found in 9.4% (10/106) patients; and accelerated phase found in 8.5% (9/106) patients (Figure 2B). The ratio between male and female in each phase are 1.8:1 for CML-CP, 3.5:1 for CML-AP and 1:1 for CML-BC (Figure 2C). The age distribution ranged between 18 and 85 years old, with the majority between 51 and 60 years old (mean, 46.8 years). Approximately 84% (156/185) patients are below 60 years old (Figure 2D).

The detection of *BCR-ABL* transcript type rearrangement was done initially by multiplex PCR because this method allows detecting several types of *BCR-ABL* transcript rearrangements in one reaction. Among 185 CML patients, 183 (98.92%) are positive for *BCR-ABL* transcript. Seventy-nine (79) of 183 samples (43.1%) succeeded to be amplified using multiplex PCR. The samples that failed to be amplified using multiplex PCR were amplified using nested PCR. The product of multiplex PCR was used as DNA template for nested PCR, and 71 samples (38.8%) succeeded to be amplified in this nested PCR. Ten (10) of 183 (5.5%) could be amplified using conventional PCR, and 23/183 (12.6%) could be amplified using nested PCR after failing to be amplified using conventional PCR.

**Figure 3:**
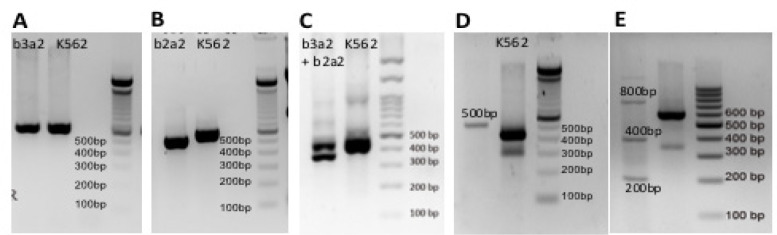
Electrophoresis of Breakpoint Types. (A), The b3a2 breakpoint type found by multiplex RT-PCR (627bp); (B), The b2a2 breakpoint type found by multiplex and conventional RT-PCR (552bp); (C), The combination of b3a2 and b2a2 breakpoint type by nested PCR, either after multiplex or conventional RT-PCR (443bp for b3a2 and 368bp for b2a2). (D) Uncommon fragment found approximately at 500bp; (D), combination of fragments approximately at 800bp, 400bp and 200bp. The band at 400bp was presumably a fragment at about 380bp and possibly considered as a rare type b2a3 (e13a3).

The majority of transcript type in the 183 patients with BCR-ABL positive was major b3a2 detected in 74.3% (136/183) patients (Figure 3A), followed by major b2a2 (41/183; 22.4%) (Figure 3B). By multiplex PCR, major b3a2 type was detected as a band at 627 bp, similar to the positive control from K562 cell line (Figure 3A), and major b2a2 type was detected at 552bp band (Figure 3B). Two samples (1.1%) showed co-expression of b3a2 and b2a2 (Figure 3C), and 1 sample (0.6%) showed band at 500bp (Figure 3D). Three samples (1.6%) samples showed multiple bands approximately at 200bp, 400bp and 800bp (Figure 3E). The band at 400bp was presumably a fragment at about 380bp. This band possibly considered as a rare type b2a3 (e13a3). All of the uncommon bands found in this study still need further confirmation. The frequency of each breakpoint can be seen in Table 1. 

**Table 1:** Frequency of BCR-ABL Breakpoint Type and Other Fragments

Breakpoint types and other fragments	Number of patients n = 183	Percentage
b3a2	136	74.30%
b2a2	41	22.40%
b3a2 +b2a2	2	1.10%
500bp	1	0.60%
800bp + 400bp + 200bp	3	1.60%

## Discussion

The initial detection of BCR-ABL transcript types in this study was done by using multiplex RT PCR method that can amplify more than one target sequence by including more than one pair of primers in the reaction (Elnifro et al., 2000; Goh et al., 2006; Mir et al., 2015). Multiplex PCR was selected as the initial method in this study because several BCR-ABL transcript types were recognized. The first multiplex PCR done in this study used a set of primers containing 2 forward primers and 1 reverse primer that can amplify 2 major transcripts (b3a2 and b2a2). Therefore, multiplex PCR has a prospect to save time and effort within the laboratory without compromising test utility. Multiplex PCR has been successfully applied in many areas of nucleic acid diagnostics, including gene deletion analysis, mutation and polymorphism analysis, quantitative analysis, and RNA detection (Elnifro et al., 2000). However, multiplex PCR has several difficulties, including low sensitivity or specificity, and poor specific target amplification. The existence of more than one pair of primers increases the possibility in amplifying unspecific products, predominantly due to the primer dimer (Elnifro et al., 2000; Markoulatos et al., 2002).

Eighteen percent (18%; 33/183) of samples failed to be amplified, and those 33 samples were then amplified by conventional RT PCR methods using a pair of primers. The conventional PCR method that was initially performed used a pair of primers for major breakpoint (M-bcr), since according to previous finding the proportion of M-bcr in patients with CML is >95% (Goh et al., 2006; Anand et al., 2012). The sensitivity of RT PCR for Ph positive cases before therapy is 97% (Cox et al., 1998). The sensitivity of multiplex RT PCR is similar with fluorescence in situ hybridization (FISH) in detecting low levels of BCR-ABL cells after hematopoietic stem cells transplantation (HSCT) (Colleoni et al., 2000; Haidary et al., 2019). In addition Goh et al., (2006) found that the sensitivity of the 1st RT PCR (conventional) result was similar to FISH, but the 2^nd^ RT PCR (nested PCR) has higher sensitivity than FISH (Kim et al., 2002).

Approximately 99% of CML patients in this study are BCR-ABL positive, with the majority being major b3a2 breakpoint (74.32%) followed by major b2a2 breakpoint (22.34%). This results of this study are similar to several previous studies that showed the frequency of CML with BCR-ABL positive are varied, but generally >95% (Goh et al., 2006; Anand et al., 2012; Hansfstein et al., 2014). The majority is major b3a2 breakpoint (Goh et al., 2006; Anand et al., 2012). Other breakpoint types were observed in this study in small proportion. Co-expression of major b3a2 and b2a2 was observed in two patients while both co-expression of major b3a2 and a fragment at 500bp were also observed in one patient. Co-expression of b3a2 and b2a2 probably is due to alternative splicing, and the clinical course is different from classic CML (Goh et al., 2006). In a different study, the fragment at 500bp was sequenced and confirmed as a b3a2 type (Sholikah et al., 2017). The fragment at about 380bp observed in this study was presumably the rare type b2a3 (e13a3). Fragments other than major b3a2 and b2a2 breakpoint found in this study still need further confirmation. 

Contrary to our results, a study in Ecuador showed that ~95% of CML patients with BCR-ABL have major b2a2 breakpoint and only 5.4% are major b3a2 breakpoint. These results suggest that it may be due to a different genetic background in the population, and a survey of the BCR-ABL transcript variants and their frequencies in different ethnic groups is needed (Hanfstein et al., 2014). Findings in Japan, Thailand and Korea were closely similar with this study in terms of the proportion of major b3a2, b2a2 and other types, which are about 60-70% for b3a2, 20-35% for b2a2 and <5% for each of the other types (Ito et al., 2004; Goh et al., 2006).

CML patients usually are diagnosed unintentionally, by complete blood cell count and blood smear. Beside hematological examination, the ideal diagnosis of CML involves FISH for t(9;22)(q34;q11.2) and quantitative reverse transcriptase PCR (qRT-PCR) for BCR-ABL that can be performed from peripheral blood of the patients (Thompson et al., 2015). But, due to some limitations, the RT PCR for BCR-ABL examination in this study was performed qualitatively. However, qRT PCR methods are proposed for future tests. 

Treatment response can be examined using the hematological approach, but the most sensitive method for monitoring CML disease is by using cytogenetics for measuring the frequency of Ph-positive cells and molecular measurement of BCR-ABL transcript levels (Baccarani et al., 2006; Jabbour et al., 2008). When a patient has achieved complete cytogenetic response (CCyR), cytogenetic evaluation is less useful for monitoring residual disease, due to the minimal Ph-positive cells that can be detected using this approach. In this situation, the presence of the leukemia-specific *BCR-ABL* gene can be monitored using the molecular approach, particularly quantitative RT PCR to quantify the level of *BCR-ABL* mRNA in peripheral blood (Jabbour et al., 2008). Subsequent long-term follow-up established the cytogenetic and molecular approach as substitutive markers of clinical outcome, residual disease monitoring, and treatment resistance. Cytogenetic and molecular approaches are now considered to be fundamental endpoints in contemporary clinical trial design for CML therapy and are essential tools guiding the choice of initial therapy in CML patients (Vigil et al., 2011).

Serial measurement of BCR-ABL transcript level is an extremely valuable method in treatment response monitoring. In Indonesia where the quantitative approach is currently unavailable, 2 step-nested amplification methods with internal primer can be used for monitoring to identify the presence or absence of BCR-ABL transcripts (Hughes et al., 2006). This 2-step combination of conventional and nested PCR has higher sensitivity compared to the single step. Therefore, it can be used to detect the low level of BCR-ABL transcript in peripheral blood.
